# What do others think? The why, when and how of using surveys in CBT

**DOI:** 10.1017/S1754470X22000393

**Published:** 2022-09-23

**Authors:** Hannah Murray, Alice Kerr, Emma Warnock-Parkes, Jennifer Wild, Nick Grey, David M. Clark, Anke Ehlers

**Affiliations:** 1 Department of Experimental Psychology, University of Oxford, United Kingdom; 2 Oxford Health NHS Foundation Trust, United Kingdom; 3 King’s College London, United Kingdom; 4 Sussex Partnership NHS Foundation Trust, United Kingdom; 5 University of Sussex, United Kingdom

**Keywords:** CBT, cognitive therapy, behavioural experiment, survey

## Abstract

Surveys are a powerful technique in cognitive behavioural therapy (CBT). A form of behavioural experiment, surveys can be used to test beliefs, normalise symptoms and experiences, and generate compassionate perspectives. In this article, we discuss why and when to use surveys in CBT interventions for a range of psychological disorders. We also present a step-by-step guide to collaboratively designing surveys with patients, selecting the appropriate recipients, sending out surveys, discussing responses and using key learning as a part of therapy. In doing so, we hope to demonstrate that surveys are a flexible, impactful, time-efficient, individualised technique which can be readily and effectively integrated into CBT interventions.

## Introduction

Clinical experience suggests that surveys can be a powerful cognitive change technique in cognitive behavioural therapies for a range of psychological disorders. They are usually used to address appraisals which cause distress and maintain symptoms, by testing beliefs, normalising symptoms and experiences, and accessing compassionate perspectives. Although a recommended part of many CBT interventions (Bennett-Levy *et al.,* 2004), they have been little researched or written about. Furthermore, there is evidence that they may be an under-utilised intervention (Waller *et al.,* 2012). For example, we asked CBT therapists participating in a post-traumatic stress disorder (PTSD) training course which CBT techniques they used in their current clinical practice. Around a third (33.12%) reported never using surveys (*n* = 311; authors’ unpublished data) when treating PTSD with CBT. This may be linked in part to practical barriers facing therapists, lack of guidance or confidence in how to carry out a survey, concerns about unhelpful responses, or other therapist cognitions that surveys are not helpful and are too time consuming. In this paper, we hope to provide a convincing rationale for why surveys can be helpful and in what circumstances, before providing a step-by-step guide to developing an effective survey together with a patient. We also provide a trouble-shooting section that addresses relevant therapist cognitions. Case studies are used to illustrate survey development; full versions of these example surveys are provided as Supplementary material.

## Why are surveys helpful?

A range of techniques are used in CBT to address important personal meanings, including by finding evidence that disconfirms negative beliefs or that supports new, more adaptive beliefs. Behavioural experiments are a core technique in CBT as a way of gathering this information experientially; a powerful way of learning not only cognitively but by experiencing changes emotionally and behaviourally (Bennett-Levy, 2003).

Surveys are a form of behavioural experiment, allowing the testing of particular beliefs linked to key emotions such as fear, shame, guilt, anger, and so on, by inviting feedback and opinions from a range of respondents. Due to the avoidance behaviours characteristic of most psychological disorders, many patients understandably hold back from discussing their beliefs with other people, especially when they feel ashamed. As a result, they are not exposed to any corrective information, for example that other people would not judge them as harshly as they are judging themselves. As surveys are usually done anonymously, confidentially and supported by a therapist, they allow for other opinions to be sought and sampled in a safe, non-exposing way, breaking this cycle of avoidance. Other times, avoidance has not been deliberate, but patients have made assumptions or been incorrectly informed about topics that they have never had the opportunity to learn about further. Surveys provide a psychoeducational tool for learning more about a range of subjects. Patients also commonly project their own negative thoughts into the minds of others, assuming that others will think the same way as them. Discussion techniques are limited in accessing the minds of others, whereas surveys allow a window into what others really think.

Surveys can be designed quickly in session and sent out easily using free online resources. Given the significant impact they can have on an individual’s negative beliefs (in our experience), they are a time efficient and highly flexible clinical strategy. Therapists are often surprised how emotive and powerful it can be for the patient to read survey responses. The learning from surveys often leads to significant belief change and can act as a springboard for the patient to make behavioural changes in therapy, like carrying out behavioural experiments they might otherwise have avoided.

## When can surveys be used as part of CBT?

Surveys can be used at any point in therapy where it would be helpful to gather more information or a range of viewpoints on a topic relevant to the appraisals that maintain the patient’s problems. As with other behavioural experiments, this typically follows the development of a shared formulation and identification of the key maintaining beliefs and processes. The over-arching goal of most surveys is to gather information about a patient’s specific belief(s). This evidence may then weigh against a negative appraisal and/or to build evidence for an alternative perspective. Here are some examples of when a survey might be useful.

### Normalising a symptom or experience

Surveys can be used to normalise symptoms or experiences which the patient is concerned about and can address catastrophic interpretations such as ‘I am going crazy’ or ‘I am permanently damaged’. For example, symptoms such as dissociation and hallucinations can feel frightening to patients but are actually extremely common, especially in certain situations such as following prolonged sleep loss or after traumatic events. Intrusive thoughts and images (such as jumping off a high place or harming someone vulnerable), often causing high levels of distress and forming part of problems such as obsessive-compulsive disorder (OCD), are a regular occurrence in non-clinical participants (over 90% in some studies, e.g. Radomsky *et al.,* 2014). Sharing this normalising information from surveys can form an important first step of exploring alternative explanations for such experiences.

Often normalisation is linked to the testing of an appraisal. For example, a patient who believes themselves to be a bad mother for experiencing post-natal depression and not feeling instantly connected to their child found it helpful to hear from other women who have a similar experience and realised that this is not evidence of being a bad parent. Another common example are intrusions in grief.

*Lena came for treatment following the death of her husband. She told her therapist that she often saw him sitting in his favourite chair or heard his voice around the house that they had shared for 20 years. Lena felt as if she was losing her mind. Lena’s therapist helped her carry out a survey to find out how common these experiences were following a bereavement. To Lena’s surprise, many of the respondents also reported seeing or hearing their loved one after their death. They described a range of responses to this experience, with some finding it disturbing and others reporting it was a comfort or a way of keeping their memory alive. Lena found this information helpful and began to feel less distressed when she saw her husband*.

### Discovering what behaviour is typical for others

A survey can be helpful to develop a benchmark for what is typical for other people, especially when a patient has had long-term experiences of thinking or behaving in a particular way. Surveys can be a first step in shifting patients’ appraisals about threat and their linked safety behaviours. For example, someone with OCD may use a survey to find out how much time other people spend on certain tasks which they do to excess, such as cleaning, handwashing or checking. Someone with PTSD following repeated exposure to a high threat environment (such as a domestic abuse survivor or military veteran) may use a survey to better understand how much caution others take in certain situations where the patient has historically needed to be extremely careful but needs to update their benchmark for an appropriate level of caution now that they are out of danger.

*Hamid experienced panic attacks where he believed he was having a heart attack. He began using many health-related checking behaviours including taking his pulse and blood pressure several times a day. He also avoided activities that might cause his heart to race such as exercising too hard, as he feared it could trigger a heart attack. Hamid conducted a survey to see whether other people used similar precautions and was surprised to learn that most people did not, and in fact believed that deliberately increasing their heart rate was a healthy strategy to reduce, not increase, the chance of a heart attack. This encouraged Hamid to try a behavioural experiment with his therapist where they both deliberately increased their heart rates by running on the spot to test if a heart attack was triggered*.

### Testing the water

A survey can be useful to ‘test the water’ and discover more information before then deciding a next step, such as disclosing an experience to others. It can also be used to seek opinions on a physical feature, such as scarring, that a patient usually hides, to assess how people would respond. This can lay the groundwork for further behavioural experiments, such as revealing a secret to a trusted person or allowing scars to be visible in public and observing responses.

*Jenny was raped as a teenager by an older boy at school. At the time, she pretended to herself that it hadn’t happened and didn’t tell anyone. Many years later, she developed PTSD, triggered by the boy (now an adult) contacting her on Facebook. In treatment, Jenny told her therapist that she was still afraid to tell people about the rape, believing they might think she had somehow invited it or that she should have fought back (Jenny froze at the time). They agreed to carry out a survey, asking people what they would say to a friend who disclosed this experience. The responses were universally supportive, with no one blaming the victim in this scenario (all agreed the perpetrator was 100% to blame), nor endorsing Jenny’s beliefs that she had invited it or should have fought back. The survey results encouraged Jenny to take the risk of disclosing to a close friend that she had been raped. Her friend was compassionate and supportive*.

### Testing how other people judge one’s appearance, behaviours, symptoms or past experiences

Surveys can be particularly powerful tests of a patient’s beliefs about how others judge them. Concerns focus on appearance, behaviours, symptoms or past experiences.

*Duncan was receiving CBT for social anxiety disorder. He was extremely anxious about blushing, which he had been teased about as a child. He believed that people would think he was effeminate, weak, and pathetic if he visibly blushed, or that women would think he fancied them. As a result, if he began to feel hot and thought he was blushing, Duncan would leave a conversation and walk away. Duncan’s therapist helped him construct a survey to ask people’s opinions about blushing, what they thought of people who blushed and if they ever blushed themselves. No one endorsed Duncan’s predictions that they perceived blushing as signs of being effeminate, weak, pathetic, or fancying someone. Most people ascribed other causes to blushing, such as a person being hot, excited, or embarrassed, and everyone reported blushing themselves. Many people also saw it as a positive and endearing feature in others. The survey gave Duncan the confidence to try further behavioural experiments including staying longer in conversations if he felt himself blushing and watching for signs that others responded no differently to when he didn’t feel hot*.

Surveys are powerful tools to discover that other people are less judgemental, and more understanding and compassionate than the patient expects, such as in Jenny’s example. This is especially helpful if a patient is experiencing self-critical thoughts or shame. Many patients with low self-esteem and/or high shame struggle to access self-compassion, particularly where shame has been longstanding, or they have significant experiences of emotional abuse. Receiving compassionate responses from a group of strangers (who presumably are not motivated to be disingenuous) can be hugely powerful in demonstrating that not everyone will hold the same critical stance as the patient or past abusers.

*In treatment for depression, Piotr described self-critical thoughts and a long history of low self-worth linked to emotional abuse from his mother in childhood. As an adult, he had experienced two long periods of unemployment related to his depression and his marriage had failed after his wife was repeatedly unfaithful. Piotr believed that these experiences showed that he was ‘a loser’ and ‘useless’. Even though his friends had been supportive, Piotr believed that they secretly saw him as a failure. In his survey, Piotr described this history and asked people to be truthful in their responses. He asked whether respondents would view a man with these experiences as ‘a loser’ and ‘useless’. At the recommendation of this therapist, Piotr included questions about what respondents would say to a good friend with the same history and whether they themselves, or someone close to them, had experienced unemployment or divorce. This prompted compassionate responses, and many respondents shared their own experiences of unemployment and relationships breaking down. Piotr selected some of the compassionate responses and saved them on his phone, looking at them regularly when his thinking became self-critical to remind himself that other people would not judge him as harshly as he judged himself*.

## How to design an effective survey

This section focuses on the nuts and bolts of how to do a survey, including who and what to ask, how to ask, and how to collate feedback and discuss it with patients. These suggestions are summarised in Table 1.

### Who to ask?

It is important to be mindful of who to approach to ensure responses are as meaningful as possible for patients. Collaboratively discussing who to send the survey to may help reduce the chances of the responses being discounted. Asking the patient about who they think would be most helpful to ask is the first step, without making assumptions. Most patients are happy for the survey to be sent to anyone, but some may wish to hear from specific groups, so considering culture, ethnicity, race, heritage, sex, identified gender, age, LGBTQI+, people with/without disabilities and socioeconomic status is important. For example, a new father experiencing intrusive thoughts about harming his baby is more likely to be interested in hearing about other parents’ experiences of intrusive thoughts, or perhaps only other fathers’ responses. Another example might be a young Asian woman who feels stigmatised for having been sexually assaulted who may be most interested in the views of people with Asian heritage. It is important to include a range of people, rather than only therapists, to avoid the common dismissal, ‘yes, but they are just being nice; they are all therapists’.

Gaining consent to send the survey more widely, while confirming confidentiality and anonymity, is key. As a rough estimate, sending the survey to 10–12 people with the aim of getting 8–10 responses provides enough data without the risk of overwhelming the patient. We also recommend encouraging the patient to complete the survey themselves, being as objective as possible (imagining they are responding about someone else) and/or asking for the patient’s predictions about what others will say.

### What to ask?

Surveys may purely consist of questions, present a brief vignette based on the patient’s experience with associated questions, or feature pictures with linked questions. Questions must link to the formulation and specifically target key cognitions and their meanings. Surveys are best kept clear, brief and succinct; restricting the number of questions will encourage as many responses as possible. We usually aim for at least five, but definitely less than ten! It will also help to limit the time required to devise the survey in session – developing a brief survey need only take 10–20 minutes.

Questions are written together in session as collaboratively as possible. Therapists can assist with suggesting or shaping questions so that they target the patient’s specific beliefs. In general, we start with open questions (e.g. ‘why do you think people stutter?’) and then use more specific questions (‘would you think badly of someone who stutters?’) directly related to patients’ negative beliefs. The open questions often reveal that people have a range of different views about the things that patients are concerned about. The final closed questions often show that others rarely confirm patients’ negative interpretations. In order to ensure that the survey is convincing, it is useful to encourage respondents to write detailed answers, not just yes/no responses. Belief ratings (0–100) can also sometimes be useful.

It can be helpful to ask questions about others involved, where relevant. For example, where interpersonal trauma has occurred, it can be helpful to ask peoples’ opinions of the perpetrator(s). For other problems it can also be helpful to gather perspectives on the behaviour of other people towards the patient, e.g. ‘What would you think of someone who was very critical of a person for stumbling over their words?’. This is often something the therapist may need to suggest, particularly where there is excessive self-blame. Finally, we recommend adding in a question towards the end of the survey designed to elicit direct compassion for the patient, such as ‘what would you say to a friend or a loved one who faced this situation?’.

### Questions only

Questions without any contextual information can be useful for normalising, psychoeducation or fact finding. This might be helpful in panic disorder, e.g. *Have you ever felt surreal or like you are in a dream? How often does this happen to you? When it happens, what do you make of it? How much do you worry that it means you are about to go mad or lose control? Please rate 0–100, where 0 is not at all and 100 is extremely, and please comment on your answer*. Or in OCD, we could ask: *How often do you wash your hands during a typical day? In what kinds of situations do you wash your hands? Do you wash your hands before taking clean laundry out of the washing machine? How long do you spend washing your hands typically in seconds or minutes?*

### Vignette with associated questions

Vignette-based surveys are used when it would be helpful for the respondents to have some context to the patient’s experience in order to answer the survey questions. The vignette is developed collaboratively with the patient, using a pseudonym. It is important to focus on the patient’s key appraisals and keep the survey concise. We usually preface the survey with a sentence about the nature of the situation described, so that respondents can choose not to read on if they may find the content distressing. Vignette-based surveys are helpful in targeting shame, guilt or anger-related beliefs in PTSD, e.g. asking about how much someone was to blame for their role in a road traffic accident (guilt), or whether they would think someone was disgusting for wetting themselves during an attack (shame).

*Kamal had been assaulted and blamed himself for not fighting back, believing this showed he was a weak and cowardly person. Kamal’s therapist helped him write a vignette, using the pseudonym ‘Sajid’ to introduce his survey: ‘Sajid was attacked by a group of four men on his way back from work. They took his wallet and phone and then punched him. They pushed him to the floor and kicked him many times. Sajid did not fight back. He tried to curl into a ball and protect his head. After a while, they left. Sajid was badly bruised and had a fractured cheekbone after the assault.’*

Again, we start with open questions following a vignette, e.g. *‘What are your general impressions of what happened to Sajid?’,* followed by specific questions that address particular meanings: e.g. *‘How much do you believe Sajid was weak and cowardly for not fighting back during the attack (0–100%)?*; questions about the perpetrators, e.g. ‘*What are your impressions of the attackers?’* (general), *‘how much do you think the attackers were to blame for the assault (0–100%)?’* (specific), ‘*how much do you think they were weak or cowardly for attacking Sajid (0–100%)?’* (specific); and a final question to prompt compassion, e.g. *‘If Sajid was your friend and told you about the attack, what would you say to him?*’.

### Pictures with linked questions

Photo-based surveys can be helpful where patients have beliefs about visible characteristics, e.g. scarring, perceived body defects or disabilities. Again, it is vital to target the meaning and beliefs about these characteristics in the survey. For example, asking people for their impressions of someone’s face which features a scar, then pointing out the scar and asking directly what they make of it can help patients discover that (a) the scar is not as noticeable as they feel it is, and (b) even if someone does notice, they don’t all think it is ‘weird and ugly’ like the patient may predict, nor would they think about it for long or remember it.

*Tosin suffered with body dysmorphic disorder. He had a distorted image of the shape of his profile, believing he looked disfigured and disgusting, and that other people would think so too. He and his therapist devised a photo survey including a photo of Tosin’s profile alongside a number of other profile images taken from Google Images, asking people which (if any) images they thought were disfigured or disgusting*.

Pictures may have other uses as part of surveys, for example, asking opinions on a scenario which is best illustrated visually.

*Patricia suffered with post-natal depression and was ashamed that her house was so messy since having her second child. This prompted a lot of negative thoughts, self-criticism and social withdrawal. Patricia and her therapist shared a photo of her lounge covered with toys in a survey to other new parents. She discovered that others reported their house looked exactly the same and that they would not judge her negatively. Some respondents even kindly shared a photo of their own home in response to normalise Patricia’s experience*.

### Ready-made surveys

The OxCADAT resources website (https://oxcadatresources.com/) shows a range of street video surveys regarding opinions about common concerns held by people with social anxiety disorder, e.g. stumbling over words, blushing and feeling boring at https://oxcadatresources.com/social-anxiety-disorder-training-videos/. Purdon and Clark (1993) provide a list of common types of intrusive thoughts in non-clinical participants which can be useful in normalising their occurrence for people with OCD. Similarly, there are visual surveys that help to normalise body appearance, e.g. the Great Wall of Vagina by Jamie McCartney is a sculpture of 400 women’s vulvas which normalizes the variation in shape and visual appearances in women’s genitals which could be used to address beliefs about appearance following childbirth or longer-term body image problems. Such resources can be accessed during therapy sessions to help the patient view how others see their concerns and to help explore and challenge their beliefs.

There can also sometimes be value in a therapist carrying out a general survey on a topic without input from the patient, or sharing responses from relevant past surveys they have conducted (with personal or identifiable details removed) to provide an initial window into the perspective of other people, particularly when a patient is reluctant to devise their own survey. However, individualised surveys should ideally follow, as each patient’s beliefs are idiosyncratic, so greater insight is usually achieved when their specific, personalised questions can be included.

### How to get responses?

The easiest and most reliable way of obtaining anonymous, confidential, honest and open responses is to use online platforms, such as Survey Monkey or Google Forms. Ideally, both therapist and patient will send the survey to people they know. If appropriate, audio or video responses may be more emotionally powerful than written responses. These can’t be collected using the same online platforms but can be recorded on the patient or therapist’s phone and saved in an online folder.

### How to collate feedback

It can be helpful to collate responses so that all answers to each individual question are grouped together to enable maximum learning to each question. It is essential to read through responses *before* giving them to patients to be aware of the range of responses. There will likely be variation in opinions, particularly where a broad audience has been approached, and this can be helpful and representative of the general population’s varying viewpoints. See the ‘Trouble-shooting’ section below for how to deal with responses that support the patient’s predictions.

### How to share and use responses

Try to keep survey reviews as active as possible: encouraging the patient to make predictions about the responses before looking through them and then updating the predictions. This is similar to using a behavioural experiment record form. If they have completed the survey themselves, comparing their responses to other people’s, can also lead to important learning (e.g. that they are more critical of themselves than others are, which may be true more generally as well). It is helpful if patients read responses aloud (unless surveys are particularly long/have many respondents!) to enable reflection on key points. Guided discovery is important when looking at responses: asking what patients made of individual and overall responses and what they have learned about their predictions and in turn their beliefs, re-rating their original predictions as part of this process.

As with all cognitive and behavioural interventions, writing down key learning points is essential (Padesky, 2019). It is then important to discuss how the patient will build on these learning points and take them further, e.g. the survey may be the first step towards telling family members or trusted friends about their particular beliefs, or it could be to start reducing the number of times they spend washing their hands each day in line with other people surveyed, setting up a behavioural experiment to further test beliefs, or providing key information to bring into the updated reliving of a particular hotspot in PTSD. Flashcards of key information can also be used between sessions. There is a video example of preparing and feeding back a survey with a PTSD patient on https://oxcadatresources.com/surveys/

## Trouble-shooting

### I have short/limitedsessions — I don’t have time to do a survey

Surveys need not be very time-consuming, especially once therapists get more confident with the technique and adept at using online survey tools. We often find that a quick survey can take only 10–20 minutes to devise in session, with similar time required to add to an online system and generate a link which can be emailed to colleagues and friends (and to the patient to share if they so wish). Filling in the survey themselves and/or making predictions can be set as a homework task. Discussing the findings in the following session is the next step and may take most of a 60-minute session to fully explore new learning in terms of the patient’s beliefs and plan how to take it forward. CT-PTSD sessions are typically 90 minutes, leaving enough time for additional tasks, such as integrating new information from the survey into memory updates. As we often find that the findings from surveys can be very powerful, we see this as a worthwhile use of session time. It is often experiential techniques like surveys which lead to the biggest cognitive and emotional shifts in treatment and are memorable to patients after therapy ends. As with other behavioural experiments, therapists can seize the opportunity to address key beliefs experientially when they arise in session – these are often more time-efficient interventions than other forms of guided discovery.

### My patient is reluctant to try a survey

Patients may have blocking beliefs about surveys which can be identified and addressed in session. For example, they may have concerns about anonymity or negative responses. These may interact with our own concerns as therapists, leading to us holding back. Many concerns can be addressed through mutual agreement about how the survey will be set up (e.g. using an anonymous platform, pseudonyms, sent out by the therapist). We acknowledge that we cannot know the results in advance, and this introduces the risk of some responses that confirm the patient’s appraisals. However, this is rare, and the therapist will be there to support the patient with any upsetting responses. Using ready-made surveys on similar topics if available can also be useful as a precursor to illustrate common responses. In our experience, most patients (and therapists when they are new to the technique) are surprised by how helpful surveys can be.

### Some of the responses supported my patient’s beliefs

With responses that support the patient’s predictions, we can help our patients to formulate and view them in context, e.g. one out of 15 people said it is a sign of weakness to blush – 94% said it was not a sign of weakness. What do you make of that? Do most people judge blushing negatively or is this a rare opinion? What do you think about the one response linking blushing to weakness – does it say more about you or them? Is it possible this person had some anxiety about this topic themselves? Similarly, if three out of 12 people said they would think a friend who had been raped is ‘damaged’, it would be important to consider their comments (hence why we ask respondents to comment on their answers), as they might qualify their responses with a sense that their friend would need help and support to overcome their reactions to being raped rather than being a *damaged person* per se. Discussing any concerns or questions in supervision can be helpful before sharing with the patient.

### My patient has discounted the results

Patients sometimes discount the results of surveys, for example saying that people are just being nice, or the results are not representative. There may be some thinking errors at play here which can be addressed through Socratic questioning, for example considering evidence for and against people being dishonest in their responses to an anonymous survey, and asking the patient to imagine they are reading another person’s survey results – would they still think they were biased? It can also be helpful to repeat the survey with different questions or distribute it to a different group if it has not fully addressed the patient’s appraisals.

## Conclusion

Surveys are a flexible, time-efficient and impactful strategy to address patients’ beliefs about a range of topics across various psychological disorders. Designed collaboratively between the therapist and patient, they can be sent out anonymously and confidentially, allowing the patient to hear viewpoints that they might otherwise struggle to access. For therapists who have concerns about using surveys in therapy (for example that they are too time-consuming, difficult to conduct, or answers may be unhelpful), we encourage them to try a behavioural experiment themselves and spot an opportunity to try a survey in treatment!

## Supplementary Material

N/A

## Figures and Tables

**Table 1:** Summary table of survey design

Survey decisions	Areas to consider
Who to ask	Agree collaboratively with patient Consider with the patient which recipients (e.g. cultural groups, relevant experience, etc.) will give most meaningful responses for them It can help to include non-therapists Ask patient to complete themselves, as if for a stranger and/or note their predictions
What to ask (all surveys)	Link to formulation, targeting patient’s specific beliefs Overall, keep questions clear, brief and succinct Start with an open-ended question, then specific questions to target beliefs Include rating scales and ask respondents to comment on their answers End with a question designed to prompt compassionate response Ask for opinions on others in the scenario as appropriate (e.g. critical others, perpetrators)
Questions only Vignette plus questions	Useful for normalising, psychoeducation or fact finding Useful for surveys targeting guilt, shame, anger Develop a brief account of scenario using a pseudonym with patient Ask for general opinions on the scenario, then specific questions to target beliefs
Pictures plus questions	Use photo-based surveys for beliefs about visible characteristics, e.g. scarring, distorted self-images, or where photos help illustrate the subject matter Target the meaning and beliefs of these characteristics in the survey
Ready-made surveys	Helpful adjunct to individualised surveys
	For example, street surveys for social anxiety disorder at https://oxcadatresources.com/social-anxietv-disorder-training-videos/ or visual surveys to normalise body appearance, e.g. the Great Wall of Vagina Can be accessed during therapy sessions Should not replace individualised surveys
How to get responses	Online platforms, e.g. Survey Monkey or Google Forms. Ideally, both therapist and patient send to people they know Audio or video responses can be powerful if possible
How to collate feedback	Collate responses so that all answers to each individual question are grouped together if possible Read through responses *before* giving them to patients to prepare for range of responses Discuss any concerns or questions in supervision before sharing with the patient
How to share and use responses	Ask patient to make rated predictions before looking at responses and then update predictions after reading Patients read responses aloud to enable reflection on key points Encourage patients to highlight key points Use guided discovery to consider survey in relation to key beliefs Discuss how to build on learning points and take them further Save key information on flashcards

## Data Availability

Data availability is not applicable to this article as no new data were created or analysed in this study.

## Key learning aims

After reading this article, it is hoped that readers will be able to:

(1) Conceptualise why surveys can be useful in cognitive behavioural therapy.
(2) Implement collaborative and individualised survey design, delivery and feedback as part of a CBT intervention.

## Key practice points

(1) Surveys are a form of behavioural experiment which can be used in CBT for many psychological disorders to address a range of beliefs.
(2) Therapist and patient design the survey together, selecting who to send the survey to and designing questions to test particular beliefs and elicit compassionate responses.
(3) Survey results are reviewed in session, with key learning identified and taken forward in therapy.
