# Supplementary material for: What do others think? The why, when and how of using surveys in CBT
Source: Cogn Behav Therap. Author manuscript; Available in PMC 2022 Dec 14. (PMC7613932; doi:10.1017/S1754470X22000393)
Supplement: N/A [file EMS153974-supplement-N_A.docx]

**Case example surveys in full**

**Lena**

This survey is about experiences after the death of a loved one. Please only complete it if you have had this experience and are happy to answer questions about how you felt afterwards.

1. After your loved one died, how did you feel?
2. Did you notice any unusual experiences?
3. Did you ever think you had seen or heard your loved one after they had died?
4. If yes, what did you make of this experience? Did you find it distressing?
5. If a friend told you that they sometimes saw their husband who had died in the house that had shared for 20 years, would you think they were crazy? Please comment on your answer.
6. What would you say to your friend if they told you they had this experience?

**Jenny**

Please note that this survey references sexual assault. Please prioritise your own self-care and only complete it if you are comfortable to.

A young woman, Anna, was sexually assaulted as a teenager by an older boy at school. Anna had previously had a crush on the boy and had been flattered when he asked her to meet him in the park. However, she did not want to have sex with him, so when he started kissing her and trying to undress her, she tried to stop him. When he became more forceful, she became frozen in fear and did not fight back and he raped her.

1. What are your overall impressions of Anna’s experience?
2. What are your overall impressions of the boy’s actions?
3. To what extent (0-100%) do you think Anna invited the assault because she agreed to meet the boy in the park? Please comment on your answer.
4. To what extent (0-100%) do you think Anna should have fought back? Please comment on your answer.
5. How much (0-100%) do you think Anna was to blame for the assault? Please comment on your answer.
6. How much (0-100%) do you think the boy was to blame for the assault? Please comment on your answer.
7. If a friend of yours disclosed this experience to you as an adult, what would you think of them as a person?
8. If Anna was a friend of yours, what would you say to her about her experience?

**Duncan**

1. What is your gender?
2. What do you think are the main reasons that a person blushes?
3. Do you ever blush yourself?
4. If yes, in what situations do you blush?
5. If you blush, how much does it bother you?
6. When you notice someone blush, what do you think about that person?
7. If you saw a man blush, to what extent (0-100%) would you think he was effeminate? Please comment on your answer.
8. If you saw a man blush, to what extent (0-100%) would you think he was weak or pathetic? Please comment on your answer.
9. If you saw a man blush, to what extent (0-100%) would you think he fancied you? Please comment on your answer.
10. If someone close to you was self-conscious about blushing, what would you say to them?

**Piotr**

A man called Paul had a difficult childhood where his mother was mentally unwell and often very cruel to him. As an adult, he got married and then divorced after his wife was repeatedly unfaithful. Paul suffers from depression and has twice lost his job as a result.

1. What are your overall impressions of Paul?
2. How much do you think Paul is a loser because of his experiences (0-100%)? Please comment on your answer.
3. How much do you think Paul is useless because of his experiences (0-100%)? Please comment on your answer.
4. Have you, or anyone close to you, had experiences of unemployment or divorce?
5. If so, how have these experiences affected you/the person you are close to?
6. If Paul was a friend of yours, what would you say to him about his experiences?

**Kamal**

This survey is about a violent assault. Please prioritise your own self-care and only complete it if you are comfortable to.

Sajid was attacked by a group of four men on his way back from work. They took his wallet and phone and then punched him. They pushed him to the floor and kicked him many times. Sajid was terrified and did not fight back. He tried to curl into a ball and protect his head. After a while, they left. Sajid was badly bruised and had a fractured cheekbone after the assault.

1. What are your general impressions of what happened to Sajid?’
2. How much do you believe Sajid was weak and cowardly for not fighting back during the attack (0-100%)? Please comment on your answer.
3. What are your impressions of the men who attacked Kamal?
4. How much do you think the attackers were to blame for the attack (0-100%)? Please comment on your answer.
5. How much do you think they were weak or cowardly for attacking Sajid (0-100%)?
6. If Sajid was your friend and he told you about the attack, what would you say to him?

**Tosin**

Please look carefully at the four pictures attached to this email.

1. Which, if any, of these pictures do you think appears disfigured?
2. Which, if any, of these pictures do you find disgusting?
3. Please look again at picture 2. What is your general opinion of this man’s profile?
4. In your opinion, is the profile in picture 2 abnormal? If so, in what way?
5. If you had a close friend or family member who looked like picture 2, what would you say to them about their appearance?

**Patricia**

Please only complete this survey if you have young children.

Please look at the attached photo of a living room.

1. What are your overall impressions of this room?
2. What are your impressions of the person whose room this is?
3. How much do you believe the person who lives in this house is a lazy person (0-100%)? Please comment on your answer.
4. How much do you believe the person who lives here is a bad parent (0-100%)? Please comment on your answer.
5. Would you ever allow a room in your house to become this messy? Please comment on your answer.
6. The person who lives in this house is a single mum of two young children. She struggles to keep her house tidy. If this mum was a friend of yours, what would you say to her?
